# Laparoscopic Management of a Symptomatic Paraduodenal Hernia Complicated by Ureteric Compression: A Case Report

**DOI:** 10.7759/cureus.111369

**Published:** 2026-06-23

**Authors:** Palinda Ranathunga, Gagana Ganga, Buddhika Dassanayake

**Affiliations:** 1 Surgery, Postgraduate Institute of Medicine, Colombo, LKA; 2 Genaral Surgery, Teaching Hospital Peradeniya, Kandy, LKA; 3 Surgery, Teaching Hospital Peradeniya, Kandy, LKA

**Keywords:** hydroneprosis, laparoscopic treatment, left paraduodenal hernia, paraduodenal hernias, small bowel obstruction

## Abstract

Paraduodenal hernias (PDHs) are a rare variant of congenital internal hernias that result from abnormal midgut rotation and represent the commonest type of internal hernia. They often result in intermittent or acute small bowel obstruction (SBO), but can present with rare complications. A 41-year-old male patient presented with episodic abdominal pain for eight months. There were no other associated symptoms. Imaging for suspected ureteric colic was done, and it was revealed that there were encapsulated small bowel loops in the left upper quadrant (LUQ). CT abdomen was consistent with a left PDH, with associated left hydronephrosis likely due to ureteric compression by the hernial sac, and incidental right renal calculi. Laparoscopic exploration confirmed a paraduodenal recess containing trapped jejunal loops. During the surgery, the inferior mesenteric vein (IMV) was divided to facilitate reduction, and the defect was closed. Postoperative recovery was uneventful, with complete resolution of symptoms. After two days, the patient was discharged and scheduled for a clinic review. PDH is an important, rare cause for chronic, episodic, unexplained abdominal pain. It also demonstrates that rare urinary tract complications can occur, and that laparoscopic repair with defect closure is an effective and safe management strategy.

## Introduction

Rare congenital conditions known as paraduodenal hernias (PDHs) are brought on by the midgut's malrotation during embryonic development, which causes the small intestinal loops to protrude through a peritoneal defect, close to the duodenum. Despite their rarity in the general population, PDHs account for 53% of all internal hernias, representing the commonest type. Since internal hernias alone only cause 0.2 to 0.9% of all intestinal obstructions, PDHs are an uncommon but clinically significant cause of small bowel obstruction [1].

Internal hernias are not common in the general population, despite the fact that there is still a lack of worldwide data, epidemiologically [2]. PDHs make up the majority of congenital internal hernias, being more common in men, and tend to manifest most frequently during the fourth to sixth decades of life, though cases have been documented at all ages [3].

Bowel loops in the left PDH become trapped in Landzert's fossa, an anatomical recess to the left of the fourth part of the duodenum. The recess is mostly evident when the descending mesocolon does not completely fuse with the abdominal wall in the posterior aspect. Loops along the right PDH herniate into Waldeyer's fossa, which is situated inferiorly compared to the third section of the duodenum and behind the superior mesenteric artery. These fossae's developmental abnormalities give rise to the possible hernia sac, which may subsequently manifest symptoms [4]. The anatomical boundaries of Landzert's fossa are the inferior mesenteric vein (IMV), posterior parietal peritoneum, and the left branches of the middle colic artery anteriorly, and the fourth part of the duodenum medially [5]. The relationship between the right PDH and the inferior mesenteric vessels differs, and it lacks the same vascular landmarks. Although this step carries risk, surgical repair may necessitate the consideration of dividing the IMV to facilitate reduction and closure of the defect [6].

PDH symptoms include intermittent postprandial abdominal pain, nausea, and bloating, as well as acute abdominal pain with vomiting and distension that may be a sign of small bowel obstruction. They are mostly misdiagnosed as renal colic or functional gastrointestinal disorders due to the persistent and ambiguous nature of the symptoms [7].

Due to the low sensitivity of ultrasonography and plain radiography in identifying PDHs, diagnosis is difficult [1]. The gold standard is still abdominal computed tomography (CT), which shows that the small bowel loops are covered in a clustered, sac-like structure with surrounding mesenteric vessels displaced and a consistent anatomic relationship to the duodenum. In unclear cases, diagnostic laparoscopy or magnetic resonance imaging (MRI) may be helpful [8]. PDHs can result in potentially fatal side effects, such as bowel ischemia and strangulation, if left untreated [9]. Although uncommon, extra-intestinal problems can include vascular obstruction, hydronephrosis, or ureteral compression [10].

Because of the high risk of complications in the future, surgery is the definitive treatment, even for asymptomatic patients. Although laparoscopic repair has gained popularity due to its shorter hospital stays, less postoperative pain, and lower morbidity, open surgical repair was the standard method in the past. To achieve sufficient exposure and prevent recurrence, the procedure typically involves reducing the herniated intestinal loops, closing the hernia defect, and dividing the IMV if necessary [11].

In certain cases, PDHs might mimic urological disorders like ureteric colic and induce secondary ureteric compression with hydronephrosis, presenting a rare and diagnostically complex scenario that healthcare providers must recognize.

## Case presentation

A 41-year-old male patient presented with approximately eight months of episodic abdominal pain, a history corroborated in July 2023. On examination, his temperature was normal. His blood pressure was 120/80 mmHg, and his pulse rate was 72 bpm and regular. His electrocardiogram (ECG) was normal. He had undergone a ureteroscopy a year earlier. Other than that, his past medical history and allergic history were not significant. 

An ultrasound of the kidney, ureter, and bladder (KUB) revealed a small, non-obstructive 4 mm calculus in the right kidney; the left kidney appeared normal, with no hydronephrosis. A subsequent CT-KUB was performed to further evaluate the suspected left ureteric colic (Figure 1).

**Figure 1:**
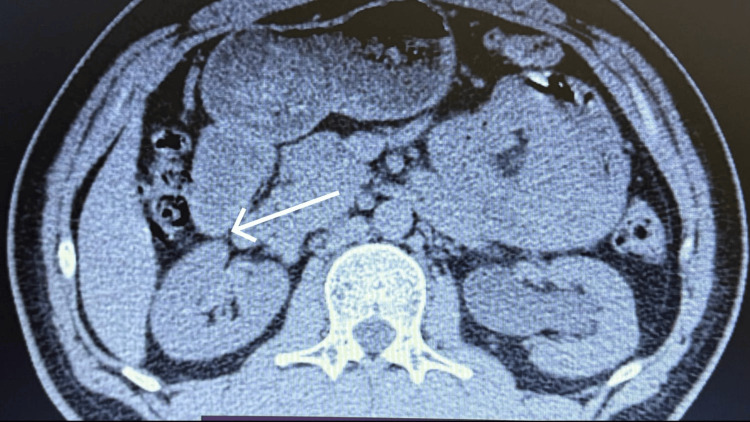
Axial non-contrast computed tomography demonstrating a dilated proximal bowel loop, with the arrow indicating the site of bowel cutoff

The most significant incidental finding was encapsulation and crowding of small bowel loops in the left upper quadrant (LUQ) consistent with an internal hernia. The size of the sac measured approximately 8×8×15 cm, along with left-sided hydronephrosis characterised by a lower polar calyceal separation of 12 mm, an antero-posterior (A-P) diameter of the intrarenal pelvis of 18 mm, and an A-P diameter of the extrarenal pelvis of 26 mm, likely due to compression of the left ureter by the hernial sac. It confirmed two small non-obstructive calculi measuring 3-4 mm, which were identified in the lower and middle groups of calyces. Blood investigations were a normal full blood count (white blood cell count (WBC): 6.0 (x 10^3^/µL), red blood cell count (RBC):5.16 (x 10^6^/µL), Haemoglobin: 14.6 g/dl, Haematocrit: 46.1%, Platelet count: 192 ( x 10^3^/µL) and coagulation screen (prothrombin time/international normalised ratio was 0.92).

Blood investigations revealed a normal full blood count. The results are shown below (Table 1).

**Table 1:** Summary of full blood count parameters

Parameter	Result	Reference Range	Units
White blood cell count (WBC)	6.0	4.0–11.0	10^3^/μL
Red blood cell count (RBC)	5.16	4.50–5.90	10^6^/μL
Haemoglobin	14.6	13.5–17.5	g/dL
Haematocrit	46.1	41.0–53.0	%
Platelet Count	192	150–450	10^3^/μL
Prothrombin time/international normalised ratio (PT/INR)	0.92	0.8–1.2	Ratio

The patient was referred to a urologist, who identified the CT findings as suggestive of a paraduodenal hernia, and later he was referred to a hepatopancreatic-biliary surgeon.

Subsequently, he underwent diagnostic laparoscopy with reduction of the paraduodenal recess under general anesthesia. Operative findings confirmed a paraduodenal recess harboring trapped jejunal loops (Figure 2).

**Figure 2:**
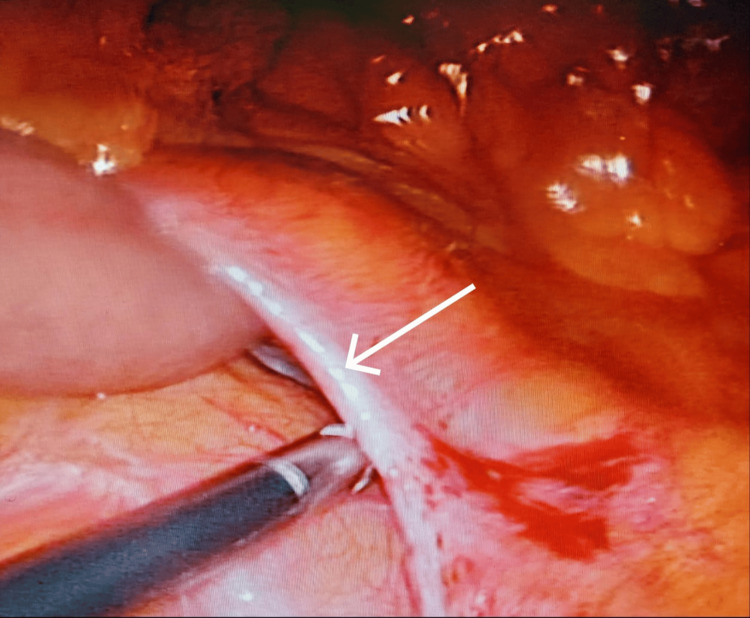
Demonstration of bowel loop herniation through the defect The arrow indicates the hernial defect.

The IMV was divided between clips and bowel loop was reduced. The hernial sac was kept wide open, and haemostasis was achieved. Subsequently, the 10 mm port was closed with 1.0 polyglactin sutures. The rest of the closure was done in standard fashion.

Postoperatively, bowel output was once a day with normal small stools, no loss of appetite, no abdominal pain. On examination, the surgical site wound was clean. The patient had an uneventful recovery and was discharged on after a month, in stable condition, with resolution of bowel obstruction. He was discharged with lactulose syrup for one month. 

The patient was followed up at the surgical clinics at the National Hospital, Kandy, after three months with investigations including serum urea, creatinine, and electrolytes, as well as a KUB ultrasound. There were no post-operative complications reported. Renal function tests were normal. Significant resolution of hydronephrosis of the left kidney was observed. However, mild persistent hydronephrosis was noted, and the patient was referred to the genitourinary clinic for further management of the renal calculi. 

## Discussion

The most prevalent kind of congenital internal hernia and a significant but frequently disregarded cause of small bowel obstruction is PDH [1]. Therefore, prompt identification is crucial because of the possibility of severe consequences like ischemia or strangulation [1]. The clinical presentation, diagnostic difficulties, and surgical treatment of a rare case of left PDH complicated by ureteral compression are discussed here, with a focus on how this case fits into and contributes to the body of existing literature.

Our 41-year-old male patient had the standard demographic reported in the literature. Males are more likely than females to be diagnosed with PDH. The male-to-female ratio ranges from 3:1 to 5:1, with a mean age of 38.5 years. According to Schizas et al., the majority of the left PDH cases manifest between the third and sixth decades of life [1]. Thus, our patient's age at presentation matches the anticipated epidemiological profile.

PDH is characterised by symptoms of acute or intermittent small bowel obstruction (SBO), nausea, vomiting, and intermittent, cramping abdominal pain [12,13]. A systematic review of 273 cases found that 82% of patients had abdominal pain, 49% had nausea or vomiting, and 30% had distension [1]. The eight-month history of episodic abdominal pain that our patient had without vomiting or significant distension fits the chronic intermittent pattern that has been reported in certain cases [14]. A mean delay of more than seven months from symptom onset to diagnosis was reported by Wong et al. [13], similar to the eight-month delay in our case. Delayed diagnosis of PDH is common. In line with most cases that happen on their own, no obvious precipitating event was found [15].

The presence of left hydronephrosis, likely due to ureteric compression from the hernial sac, was an uncommon feature of this case. There is very little literature on PDH causing hydronephrosis. The hydronephrosis was not noticed on the initial ultrasound, probably due to the intermittent nature of ureteric compression or the limits of ultrasound in detecting early hydronephrosis, underlining the diagnostic value of CT. Although PDH has been known to mimic renal colic [16], this case was an uncommon instance in which the hernia was discovered during imaging for suspected urological pathology. Since there is no known link between PDH and nephrolithiasis, the coexisting right renal calculi are probably incidental.

CT remains the gold standard for PDH diagnosis, with a reported sensitivity of 90% when classic signs are present [17]. These include encapsulated clusters of small bowel loops, displacement of mesenteric vessels, and crowding of the mesentery in the LUQ [18]. In our patient, the CT-KUB, which was done for suspected ureteric colic, revealed encapsulated small bowel loops in the LUQ consistent with left PDH. This is comparable to reports of incidental diagnosis when CT is obtained for unrelated purposes [19].

Our patient underwent diagnostic laparoscopy with reduction of the paraduodenal recess, and the IMV was divided under general anesthesia. For all diagnosed PDHs, surgical repair is recommended due to the high risk of strangulation and obstruction, which is estimated to be between 50 and 57% [1]. Studies showing shorter hospital stays, less postoperative pain, and lower recurrence rates with laparoscopic repair compared to open repair have made the former more popular. Laparoscopic reduction of the hernia with division of the IMV was performed in our case, which is in line with methods for enlarging the hernial orifice that have been reported in complex left PDHs [20]. Division of the IMV can be considered safe due to the left colon's robust collateral venous drainage via the Drummond marginal artery and the Riolan arc, which prevents ischaemia from damaging the descending colon [20]. 

The clinical team's initial diagnosis of ureteric colic was understandable given the constellation of periodic flank pain and left hydronephrosis on imaging. At this point, urolithiasis, ureteropelvic junction (UPJ) obstruction, retroperitoneal fibrosis, and extrinsic ureteric compression from an abdominal mass were among the differential diagnoses [8]. Functional gastrointestinal disorders, adhesional SBO, and irritable bowel syndrome were also considered given the episodic abdominal pain, which is consistent with the diagnostic challenges outlined by Manojlović et al.. They noted that the average time to diagnosis of PDH in the literature exceeds 21 months due to its resemblance to other conditions [8]. Khan et al. were among the first to describe PDH mimicking renal colic, highlighting the diagnostic confusion that arises when an internal hernia presents with flank pain [16]. 

Cases featuring this presentation are rarely documented in adult patients. A pediatric case of left PDH causing hydronephrosis through compression of the ureteropelvic junction was reported by Wang et al. It was only discovered intraoperatively during planned pyeloplasty, bringing out how this typical issue can result in misguided invasive interventions, if PDH is not taken into consideration in the differential [21]. 

With an early return of bowel function, no postoperative complications, and discharge on the second postoperative day, our patient recovered quickly. According to the literature, laparoscopic repair has a lower recurrence rate and a quicker recovery than open surgery, with a mean hospital stay of three to five days. The majority of authors follow patients for six to 12 months with clinical evaluation, saving imaging for symptomatic recurrence, according to Schizas et al. [1]. Follow-up recommendations vary. The primary purpose of our patient's planned follow-up visit to the urology clinic, which included renal function testing and ultrasound, was to reevaluate the hydronephrosis. Similarly, a surgical clinic follow-up was also planned.

## Conclusions

In summary, PDH can present with a significant diagnostic challenge, especially when significant bowel symptoms are absent, but there is a presence of atypical extra-intestinal manifestations such as left-sided hydronephrosis due to the ureteric compression. In cases with a high index of clinical suspicion, a CT scan should be performed to diagnose and assess the severity. Laparoscopic management is a safe alternative for this condition. Also, division of the IMV was executed safely in this case without any short-term ischemic complications till the follow-up was done, as there are robust collateral venous drainage pathways. Thus, laparoscopic management can be a safe and viable technical option in the management of a complex left PDH.
